# Do American Dippers Obtain a Survival Benefit from Altitudinal Migration?

**DOI:** 10.1371/journal.pone.0125734

**Published:** 2015-04-23

**Authors:** David J. Green, Ivy B. J. Whitehorne, Holly A. Middleton, Christy A. Morrissey

**Affiliations:** 1 Centre for Wildlife Ecology, Department of Biological Sciences, Simon Fraser University, Burnaby, British Columbia, Canada; 2 Department of Biology, University of Saskatchewan, Saskatoon, Saskatchewan, Canada

## Abstract

Studies of partial migrants provide an opportunity to assess the cost and benefits of migration. Previous work has demonstrated that sedentary American dippers (residents) have higher annual productivity than altitudinal migrants that move to higher elevations to breed. Here we use a ten-year (30 period) mark-recapture dataset to evaluate whether migrants offset their lower productivity with higher survival during the migration-breeding period when they occupy different habitat, or early and late-winter periods when they coexist with residents. Mark-recapture models provide no evidence that apparent monthly survival of migrants is higher than that of residents at any time of the year. The best-supported model suggests that monthly survival is higher in the migration-breeding period than winter periods. Another well-supported model suggested that residency conferred a survival benefit, and annual apparent survival (calculated from model weighted monthly apparent survival estimates using the Delta method) of residents (0.511 ± 0.038SE) was slightly higher than that of migrants (0.487 ± 0.032). Winter survival of American dippers was influenced by environmental conditions; monthly apparent survival increased as maximum daily flow rates increased and declined as winter temperatures became colder. However, we found no evidence that environmental conditions altered differences in winter survival of residents and migrants. Since migratory American dippers have lower productivity and slightly lower survival than residents our data suggests that partial migration is likely an outcome of competition for limited nest sites at low elevations, with less competitive individuals being forced to migrate to higher elevations in order to breed.

## Introduction

The spectacular migratory behavior of many birds, ungulates, insects and fish has fascinated humans for centuries. Migration is thought to allow individuals from migratory taxa to reduce intra-specific competition, exploit spatial and temporal variation in resources (particularly food), escape predation or parasites [1–7] and, consequently, have higher reproductive success [8]. However, migration, that may involve seasonal movements of over 20,000 kilometres is energetically costly and may increase exposure to predators, disease, and harsh weather conditions [8–11]. Studies suggest that daily mortality rates may be 6–15 times higher on migration than when sedentary on breeding or wintering grounds [12,13]. Unsurprisingly, comparative studies have found that migration has consequences for other life history traits [14–16].

Studies of partial migrants, species where some individuals migrate but others are sedentary, provide an opportunity to study the benefits and costs of migration without the need to control for phylogeny. In many of these species migrants travel relatively short distances (tens to hundreds of kilometres) but obtain similar benefits and pay similar costs as long distance migrants. For example, female elk *Cervus elaphus* that migrate to higher elevation habitat during the summer gain access to forage with higher digestibility, improving pregnancy rates and calf survival [17]. However, female elk that migrate are more vulnerable to predators during migration and on shared winter grounds, because sedentary individuals (residents) select habitat closer to humans that are avoided by predators, balancing the demographic fitness of migrants and residents [18]. In contrast, roach *Rutilus rutilus* that migrate from shallow lakes to streams during the winter obtain a survival benefit because they are less vulnerable to avian predators (cormorants *Phalacrocorax carbo*) than roach that remain in lakes year-round [19]. Migratory roach, however, pay a foraging cost [20] suggesting they trade-off safety from predation for access to food [21].

Trade-offs between productivity and survival may not be observed if the migratory strategies of partial migrants are density or condition dependent. For example, local density influences the proportion of red-spotted newts (*Notophtalmus viridescens*) that migrate from ponds to terrestrial habitat in the winter [22]. Individuals that migrate then pay a reproductive cost; migratory males return to the breeding pond with smaller tail fins reducing their mating success [23] and migratory females produce smaller larvae than residents [24]. Migrants that over-winter in terrestrial habitat also have lower winter survival than residents that over-winter in ponds [25]. Partial migration is also argued to be a conditional strategy with unequal fitness pay-offs in the European robin *Erithacus rubecula* [26]. Sex influences the propensity to migrate in this species; male robins in parks and gardens are less likely to leave their breeding grounds than males in woodlands or female robins that almost all migrate. Resident male robins had far higher survival, established a breeding territory early and had higher mating success than migratory males.

American dippers *Cinclus mexicanus* are a partially migratory species in which sedentary individuals (residents) and migratory individuals over-winter at low elevations and migrants move to higher elevations in order to breed [27]. We have demonstrated that altitudinal migration does not allow migrants to gain access to superior food resources or escape nest predation. American dippers that move to higher elevations initiate breeding later and are less likely to double brood, have similar levels of nest predation, provision nestlings a diet containing a higher proportion of invertebrates (a low quality prey item compared to fish), and fledge fewer young that have lower juvenile survival [28,29]. We have therefore argued that altitudinal migration is likely an outcome of competition for suitable nesting sites at lower elevations. Migrants may therefore be subordinate individuals that have both lower productivity and lower survival than residents. Three potential trade-offs may, however, allow migrants to offset their lower productivity with higher survival. First, migration may reduce the risk of predation during the breeding season. Dippers are prey for large steelhead trout (*Oncorhynchus mykiss)* sharp-shinned and Cooper’s hawks (*Accipiter striatis* and *A*. *cooperi*) and merlins (*Falco columbarius*) [30], all of which are more common at lower elevations in the spring/summer. Second, constraints on the number of breeding attempts that can be made at higher elevations may reduce reproductive costs and enhance subsequent survival. Three, migrants may overwinter in better habitat than residents because they do not have to defend a multi-purpose territory with a suitable nest site year round. These hypotheses predict survival differences in either the migration-breeding period when migrants and residents occupy different habitat or during the winter when migrants and residents coexist at lower elevations. We therefore examined whether migratory American dippers have higher survival than residents during the migration-breeding period or during the winter. This extends previous work using a smaller dataset examining annual variation in survival of American dippers that was unable to discriminate between models where survival of residents and migrants was equal and models where survival varied with migratory strategy [28].

Winter is known to be a critical stage for the survival of European dippers [31]; apparent survival (ϕ, the probability of surviving and returning to the sampling area [32]) rates decline as winter temperatures become more extreme [33] and are lower in years when rivers flood [34,35]. Winter conditions could alter the relative costs of occupying a multi-purpose territory compared to a season-specific territory so we also investigate whether winter temperature and river flow influences variation in the survival of residents and migratory dippers.

## Methods

### Ethics statement

Targetted mist-netting and banding of birds was carried out in accordance with Canadian Council on Animal Care recommendations and under permits issued by Environment Canada (CWS Banding Permit: 10759H; Scientific Permits: 59-03-0416; 59-04-0274; 59-05-0327; 59-06-0348). Field protocols were approved by the University Animal Care Committee at Simon Fraser University (Protocol # 661B-03 and 831B-03).

### Study area and species

We conducted this study in the Chilliwack River Valley (49°0'N, 121°4'W) situated in the Skagit Range of the northern Cascade Mountains, approximately 100km east of Vancouver in south-western British Columbia, Canada. The river’s watershed is 1230 km^2^ in area and the elevation ranges from 20 to 2500 m. The Chilliwack River flows for 43.5 km between Chilliwack Lake (640 m above sea level) and Vedder Crossing (40m above sea level) where it drains into the Vedder River. The river is characterised by a shallow gradient, a wide and channelized path (26-62m), with slow moving reaches, riffles and shallow pools. The river is fed by several lower order creeks. Of these, Centre Creek, Nesakwatch Creek, Foley Creek, Chipmunk Creek, Slesse Creek, Tamihi Creek, and Liumchen Creek are the largest. Tributaries are characterized by a steep gradient, a narrow path (3-20m), and fast-flowing water passing sheer rock-walls and abundant boulders [36]. The Chilliwack River Valley lies within the transition zone between a maritime and continental climate. The valley bottom is characterized by warm, dry summers and moist, cool winters with moderate snowfall. Average daily minimum temperatures range from 11.7°C in August to -0.9°C in December, although temperatures occasionally drop to almost -20°C in the winter [37]. Higher elevations are characterised by short, cool summers and long, cold winters with heavy and persistent snowfall. Seasonal variation in flow rates on the Chilliwack River result from spring/early summer snowmelt and intense rainfall events in the winter. Days with extremely high flow rates are most common in May, June and December to February [38].

We have demonstrated that the population of American dippers in the Chilliwack River Valley is partially migratory [36]. Migrants overwinter with sedentary dippers (residents) on the Chilliwack River and lower reaches of the major tributaries, but leave their winter territories in early spring (February—April) and travel ca. 6 km (range 2–21 km) to breeding territories on creeks and streams at higher elevations. Migrants and their offspring leave these territories at the end of the breeding season [39], perhaps moving to even higher elevations [40], but are back on their wintering territories by the fall [36]. Residents in contrast occupy a multi-purpose territory year round. Male and female residents defend their territory and exclude conspecifics during the breeding season, but are less aggressive in the winter when they tolerate the presence of conspecifics in some parts of their territory [41]. We have also demonstrated that migrants and residents rarely switch migratory strategies [28], and both migrants and residents exhibit high levels of fidelity to their breeding and wintering territories [41,42].

### Banding and monitoring of American dippers

We studied American dippers in the Chilliwack River Valley from February 1999 to November 2009. We captured 642 adult birds that we marked with a unique combination of three colour bands and a USFWS metal band. We caught adults by flushing them into 6-m passerine mist nets set up over moving water in narrow channels or on edges of the river or creeks. We caught the majority of adults at eight sites (each 2 km long; elevation range 40–420 m) located at approximately 4 km intervals along the Chilliwack River from October to March. We sexed adults captured during the breeding season based on the presence of a brood patch and their behaviour (only female American dippers incubate eggs and brood young; [30]). We were unable to sex adults captured outside the breeding season as males and females overlap in size [43].

We attempted to conduct winter censuses of the same 8 sites, shared by migrants and residents, on the main river in November, January, and March each year from November 1999 to November 2009. Censuses were routinely conducted in the first two weeks of each month by two observers who walked along set stretches of the riverbank and searched all channels and lower reaches of creeks where they met the river at each site. The closure of a bridge restricted access and delayed the January 2004 census until February 2004. During censuses we recorded all birds observed and confirmed the band combinations of all marked birds using a 10-40x spotting scope. Throughout the 10 year study, we collected additional resighting data on marked birds at low elevations when trapping birds during the winter (November—March), on the river during summer censuses conducted in May and July, and in all areas of the watershed during the breeding season (March—July). We kept a detailed history of each banded bird that recorded the date and location of first capture and every subsequent resighting.

### Climate data

We extracted data on three temperature variables and three river flow variables for each day of the period between census conducted in November and January (the early winter period), and each day of the period between censuses conducted in January and March (the late winter period). We used these data to calculate six climate variables for the early and late winter periods: 1) the average minimum daily temperature, 2) the most extreme (i.e. coldest) minimum daily temperature, 3) the number of days where the temperature did not exceed 0°C, 4) the mean daily flow rate (m^3^/s), 5) the maximum daily flow rate, and the number of days with extreme flow rates. Days with extreme flow rates were defined as days where flow was above the 99^th^ percentile recorded during the winter (November—March) over the ten years of the study. Temperature data was collected at the Chilliwack River Hatchery Weather Station (Climate ID 1101N65; [37]). Flow data for the Chilliwack River was collected at Vedder Crossing (Station ID 08MH001; [38]) situated at the eastern (downstream) edge of the study area. The three temperature variables were highly correlated (all r_p_ > 0.8) as were the three flow variables (all r_p_ > 0.8) so we conducted analyses using the minimum daily temperature and maximum daily flow variables.

### Survival analysis

We examined seasonal variation in the survival of migrants and residents between November 1999 and November 2009. We defined three periods based on the timing of the winter censuses; the migration-breeding period (early March—early November) when migrants and residents occupy different habitat; the early winter period (early November—early January) and late winter period (early January—early March) when migrants and residents coexist at lower elevations. We classified American dippers as either year round residents or altitudinal migrants. Residents occupy multi-purpose territories on the main stem of the river year round and were never observed more than 1 km from their territory. Migrants over-wintered at one of the 8 trapping and survey sites on the Chilliwack River and left in the spring (February—March) to take up breeding territories elsewhere. We only allowed individuals to enter the dataset in November after they could be identified as being a resident or migrant; earlier capture and resighting information was discarded. Individuals consequently entered the dataset as adults that had completed at least one breeding season. If we had entered individuals into the dataset in March prior to their first breeding season we would have overestimated the survival of migrants as migrants are only identified upon their return to the study area in November. We censored individuals that switched strategies (n = 7; 4 residents, 3 migrants) in March when their behaviour changed. We supplemented the resighting data from the censuses conducted in the first two weeks of November, January and March with resighting data obtained when banding birds in late Oct/early Nov, late Dec/early Jan and late Feb/early Mar. Our dataset therefore included 31 resighting periods with 10 intervals between March and November (8 months), 9 intervals between November and January (2 months), 1 interval between November and February (3 months) and 9 intervals between January and March (2 months), and 1 interval between February and March.

We estimated monthly survival of migrants and residents using Cormack-Jolly-Seber (CJS) models for live encounter data based on resightings and recaptures with unequal time intervals [32,34] using program MARK. We first defined a global model that allowed apparent survival of migrants and residents to differ and vary between each period of the study (t). We did not group individuals based on the age at first capture or sex because all birds entered the dataset as adults and the gender of many birds was not known. Previous studies on European dippers [33,35] and analysis of our data on known sex individuals [28], however, did not detect differences in survival between males and females. The global model allowed resighting probabilities to vary with migratory strategy (m) and month (month; November, January, March). More complex global models were not able to accurately estimate all possible survival and resighting probabilities.

We conducted our survival analysis in three stages by creating candidate model sets that allowed us to 1) find the most parsimonious parameterization of resighting probabilities, 2) evaluate monthly apparent survival across all time intervals and three time periods while retaining the best resighting model, and 3) assess whether winter temperature and river flow could explain observed variation in winter survival. This three stage procedure reduced the number of models in any candidate model set [34]. The candidate model set examining temporal variation in monthly apparent survival of migrants and residents allowed survival to vary across all 30 time intervals, survival to be constant in the 10 migration-breeding periods but vary across all 20 winter periods (mb_c_w_t_), survival to vary only in the summer periods (mb_t_w_c_), survival to differ between the migration-breeding period, early and late winter periods (season), survival to differ between the migration-breeding period and winter (mb_c_w_c_), or survival to be constant (.). This candidate model set included models with main effects (migratory strategy (m) and time (t, mb_c_w_t_, mb_t_w_c_, season or mb_c_w_c_)) and models with all combinations of main effects and interaction terms (n = 17 candidate models). The candidate model set examining the hypothesis that winter temperature and river flow influence survival of migrants and residents on shared wintering grounds included the top three models in the previous candidate model set (see results), models that allowed winter survival to differ from migration-breeding period survival and vary with minimum daily temperature and/or maximum daily flow, and models that allowed temperature and/or flow effects on apparent survival to vary with migratory strategy (n = 12 candidate models).

Goodness-of-fit of the global model was evaluated using the bootstrap procedure implemented in Program MARK, with c-hat being estimated using the observed deviance in the global model/mean deviance of the simulated models, the observed c-hat of the global model/mean c-hat of the simulated models and the median c-hat procedure [32]. Program MARK was then used to fit the global model and other models examining recapture and apparent survival probabilities using the logit-link function. The relative fit of each model was evaluated using Akaike’s information criterion corrected for small sample size (AIC_c_). AIC_c_ scores for each model were not adjusted for overdispersion in the data as c-hat was estimated to be close to 1 (see results). Models within each candidate model set were ranked using ΔAIC_*i*_, the difference in AIC_*c*_ scores of model *i* from that of the top-ranked model (ΔAIC_*i*_ = AIC_*i*_—min AIC) with the top model having a ΔAIC_*i*_ = 0 and models with ΔAIC_*i*_ < 2 considered to have strong support [44]. Akaike weights (*w*_*i*_), calculated as exp(ΔAIC_*i*_/2)/Σexp(ΔAIC/2), were used to provide a relative index of the plausibility of each model or group of models. We present recapture or apparent monthly survival estimates from the best-supported model in each candidate model set. We also present model-averaged monthly apparent survival estimates, where the estimate of the parameter for each model is weighted by the Akaiki weights (*w*_*i*_), and 95% confidence intervals derived from unconditional standard errors which incorporate model-selection uncertainty [44].

We use model-averaged monthly apparent survival estimates to estimate annual apparent survival for migrants and residents calculating the variance in these parameters using the Delta method [45].

## Results

The survival analysis included data from 260 individuals (146 migrants, 114 residents) that overwintered on the Chilliwack River. One hundred and sixty-six individuals (64%) were resighted in more than one period (79 migrants, 87 residents). At the extremes, one migrant was resighted 10 times between November 1999 and March 2004 and one resident was resighted 15 times between November 2001 and November 2006. Migrants (n = 4) and residents (n = 3) were both observed to switch strategies from one year to the next. The effective sample size in the survival analysis was 732.

The global model described the mark-resighting data adequately (ϕ(m*t) p(m*month), P = 0.47). The variance inflation factor, c-hat, was estimated to be 0.99 using the observed deviance/mean deviance of the simulated models and 0.69 using the observed c-hat/mean c-hat of the simulated models. The median c-hat procedure also found no evidence of overdispersion; the simulated c-hat was always greater than the observed c-hat. We therefore did not adjust for overdispersion when comparing models in each candidate set.

Resighting probabilities were best parameterized using the full model that allowed resighting probability to vary with migratory strategy and month. This model had overwhelming support (w_i_ = 1.0). Estimated resighting probabilities of migrants were consistent across months (January = 0.41 ± 0.05, March = 0.37 ± 0.05, November = 0.34 ± 0.05), while resighting probabilities of residents were higher and more variable (January = 0.59 ± 0.04, March = 0.95 ± 0.02, November = 0.59 ± 0.05).

**Fig 1:**
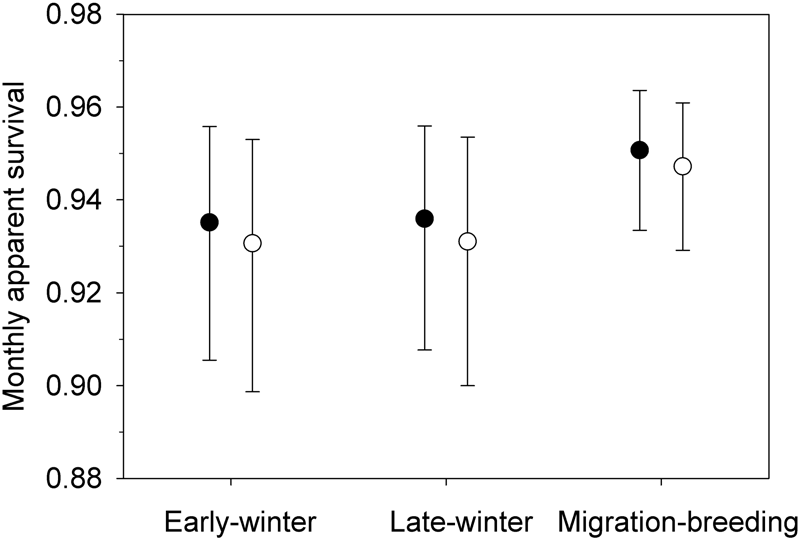
Monthly apparent survival estimates for resident and migrant American Dippers during three periods of the annual cycle. Solid dots are weighted model average survival estimates for residents; open dots are weighted model average survival estimates for migrants ± 95% CI. Weighted averages and 95% CI are derived from the five strongly supported models in Table 1.

There was strong support (ΔAIC_c_ ≤ 2) for five of the 17 models examining variation in monthly apparent survival (Table 1). Three of these five models suggested there was temporal variation in survival; the combined *w*_*i*_ of models including a temporal variable was 0.724. The best model suggested that monthly apparent survival was lower in the winter than in the migration-breeding period (winter: 0.9286 ± 0.0101; migration-breeding period: 0.9519 ± 0.0064). Two of the strongly supported models suggested that migrants had lower survival than residents, although the combined *w*_*i*_ of models including the migratory strategy variable was relatively low (0.492). Model weighted parameter estimates from the five strongly supported models show that survival is lower and more variable in the winter than during the migration-breeding period, and residents have slightly higher apparent survival than migrants in all three periods (Fig 1, S1 Table). Based on these monthly apparent survival estimates we estimated annual apparent survival of migrants would be 0.487 ± 0.032 (95% CI, 0.423–0.550) and annual apparent survival of residents would be 0.511 ± 0.038 (95% CI, 0.437–0.585).

**Table 1:** Summary of models examining survival of American Dippers in the Chilliwack River Valley.

Rank	Survival Model	AIC_c_	ΔAIC_c_	*w*_*i*_	Likelihood	K	Dev
1	(mb_c_w_c_)	1715.18	0	0.19	1	8	1040.1
2	(m+mb_c_w_c_)	1715.54	0.36	0.16	0.836	9	1038.5
3	(.)	1715.85	0.67	0.11	0.716	7	1042.9
4	(m)	1716.58	1.40	0.09	0.496	8	1041.5
5	(season)	1716.94	1.76	0.08	0.414	9	1039.9
17	(m*t)	1772.45	57.27	0.00	0.000	66	968.3

Models are ranked based on their AICc and ΔAIC_c_ with the top model having a ΔAIC_c_ = 0. We also provide the AIC weights (*w*_*i*_), the model likelihood (Likelihood), the number of model parameters (K) and the deviance (Dev). We only provide information on strongly supported models (ΔAIC_c_ <2) and the full time dependent model (m*t). Model coding: migratory strategy (m), temporal variation (t—30 periods; mb_c_w_t_—constant during the migration-breeding periods, variable during winter periods; mb_t_w_c_—variable during the migration-breeding periods, constant during winter periods; season—migration-breeding, early winter and late winter; s_c_w_c_—migration-breeding periods vs winter periods,. —constant survival during all time periods. All models parameterize resighting probabilities using p(m*month).

**Fig 2:**
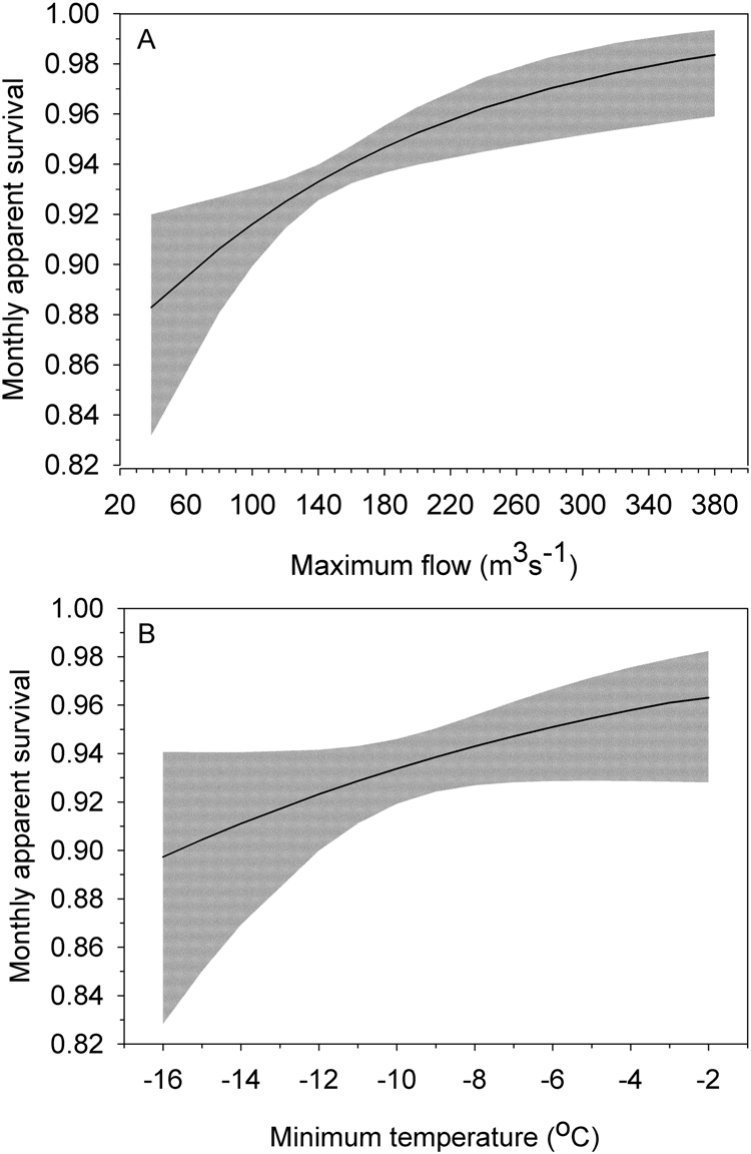
Relationship between A) the maximum daily flow rate and B) minimum daily temperature and monthly apparent survival of American dippers during early and late winter. The predictions are derived from Model 2 in Table 2. Solid lines show predicted monthly apparent annual survival when the other environmental variable is held constant at the median level observed during the study (maximum daily flow rate = 175 m^3^/s, minimum temperature = - 7.5°C). The 95% confidence intervals for each relationship are shown in grey.

Models that allowed monthly survival to be higher in the migration-breeding period than winter, and vary with river flow and winter temperature received substantially more support than models where survival simply differed in the migration-breeding and winter (Table 2). All four of the well-supported models, including the top model, contained the maximum daily flow variable. The top model indicated that monthly apparent survival during winter increased as the maximum daily flow on the Chilliwack River over each 2 month period increased (Fig 2a, effect size ± s.e = 0.0061 ± 0.0022, 95% C.I., 0.0017–0.0105). The second best model, that received a similar level of support, also included the minimum winter temperature variable. This model provided some evidence that monthly apparent survival also decreased as the minimum daily temperature decreased (Fig 2b; effect size ± s.e = 0.0798 ± 0.0456, 95% C.I., -0.0096–0.1692).

**Table 2:** Summary of models evaluating the role of river flow and temperature on winter survival of American dippers.

Rank	Survival Model	AIC_c_	ΔAIC_c_	*w*_*i*_	Likelihood	K	Dev
1	(mb_c_w_c_+maxflow	1704.95	0	0.26	1	9	1027.9
2	(mb_c_w_c_+maxflow+minT)	1705.22	0.27	0.23	0.874	10	1026.1
3	(m+mb_c_w_c_+maxflow)	1705.56	0.61	0.20	0.740	10	1026.4
4	(m+mb_c_w_c_+maxflow+minT)	1705.65	0.70	0.19	0.704	11	1024.5
10	(mb_c_w_c_)	1715.18	10.23	0.001	0.006	8	1040.1
11	(m+mb_c_w_c_)	1715.54	10.59	0.001	0.005	9	1038.5
12	(.)	1715.85	10.89	0.001	0.004	7	1042.9

Models are ranked based on their AICc and ΔAIC_c_ with the top model having a ΔAIC_c_ = 0. We also provide the AIC weights (*w*_*i*_), the model likelihood (Likelihood), the number of model parameters (K) and the deviance (Dev). We provide information on strongly supported models (ΔAIC_c_ <2) including river flow and temperature and the top three models from Table 1. Model 4 is the most complex model in this candidate set. Model coding: migratory strategy (m), migration-breeding vs winter periods (mb_c_w_c_), constant survival (.), maximum daily flow during winter period (maxflow), minimum temperature during winter period (minT). All models parameterize resighting probabilities using p(m*month).

## Discussion

Migration involving seasonal long-distance movements between continents or short-distance movements along elevational gradients or between different habitat types may allow individuals to access higher quality food or obtain refuge from predators and/or disease. In partial migrants, such as elk or roach, these benefits may be offset by costs that are experienced on migration or on shared wintering grounds [18,21] that can result in equal pay-offs for migrants and residents [18]. Alternatively, migration may be an outcome of competitive interactions with migratory individuals paying both a reproductive and survival cost of migration [25,26]. We have shown previously that altitudinal migration in a partially migratory population of American dippers incurs reproductive costs [27–29]. Here we demonstrate that these costs are not offset by survival benefits during either the migration-breeding period (when migrants move to, and return from, breeding territories at higher elevations) or during the winter periods (when migrants and residents both occupy territories at lower elevations). Furthermore, we found no evidence that migrants, that do not defend a multi-purpose territory with a suitable nest site year-round, obtain survival benefits even in winters with low river flow or cold temperatures when apparent survival is reduced. Altitudinal migration therefore appears likely to be a strategy adopted by less competitive American dippers that are unable to obtain a suitable breeding territory within the wintering range.

Predator avoidance is one of the proposed benefits of migration [3,4], and migration to areas where predators are less abundant or less effective has been found to reduce the risk of predation for ungulates and freshwater fish [17,19]. Studies of predation rates on artificial nests along an elevation gradient in Costa Rica [46], and a 350 km south-north gradient in the Arctic [6] suggest that migration may reduce predation risk for birds. In contrast we found no evidence that migration conferred a survival benefit for American dippers; the apparent monthly survival of adult dippers that moved to breeding territories on creeks at higher elevations was, if anything, lower than the monthly apparent survival of adult dippers that remained on multi-purpose territories at lower elevations. Nest predation rates are also similar at high and low elevations [27]. Dippers may be less sensitive to spatial variation in predators than other taxa because predation rates are low. Fishermen on the Chilliwack River occasionally report finding dippers inside large steelhead trout, but we have rarely observed attacks by raptors. Nests are also located on cliffs, boulders, overhanging logs and man-made structures over fast-flowing water and nest predation rates are low (ca. 16%, [27]) despite the long nestling period (22–31 days, [39]).

Winter survival of migratory and sedentary dippers was expected to differ either as a consequence of life history trade-offs between fecundity and survival [47,48] or because migrants are able to occupy the best winter habitat while residents defend permanent territories with suitable nesting habitat. Our earlier work provided limited evidence for a fecundity survival trade-off. We demonstrated that residents, all of whom attempted to breed, consistently had higher productivity than migrants [28]. We also found no difference in the physiological state of residents and migrants [41]; physiological state was assessed in the winter using size-corrected mass, hematocrit, leucocrit, total white blood cell count and heterophil to lymphocyte ratio, immunoglobulin, triglyceride and free glycerol levels, total antioxidant capacity and total oxidative status of plasma. However, we estimated that the annual apparent survival of residents was slightly, but not significantly higher, than the annual apparent survival of migrants [28]. The latter result may have been biased in favour of migrants because annual survival was calculated from one winter to the next when birds could only be classified as migrants or residents in the spring. Migrants that survived until the spring but died on migration or during their first breeding season would therefore be more likely to have been excluded from the dataset than residents, because they were less likely to be resighted and known to be alive at the time individual birds were classified as migratory or sedentary. In this study, which included data from an additional three years and minimized bias by only allowing birds to enter the dataset in the winter after they could be classified as a migrant or a resident, we found no evidence that migrants had higher survival than residents. We acknowledge that our analysis excludes young birds during the period from March to November when they migrate (or remain at low elevations) and breed for the first time. However, taken together, the physiological data and improved mark-recapture modeling suggest that year round defence of a low elevation breeding territory does not impose a significant survival cost on resident adult dippers, and that winter habitat, in areas without cliffs, boulders and bridges that provide nesting habitat, occupied only by migrants may be no better than habitat used by both migrants and residents during the winter.

Our failure to detect a fecundity survival trade-off or a cost imposed by territory defence and constraints on winter habitat selection may be a consequence of differences in the quality of resident and migratory individuals that often make life history trade-offs difficult to detect [49]. Alternatively, we may have estimated the apparent survival of migrants and residents to be equal even though the true survival of migrants is higher than the true survival of residents because migrants have lower over-winter site fidelity and are more likely to leave the study area permanently. We have shown that over the course of a single winter the site fidelity of migrants is lower than that of residents [41]. However, we have rarely observed overwintering dippers to use more than one of our eight low elevation study sites even though they are only separated by 3–40 km. Whitehorne [41] observed 4 migrants and 2 residents (total n = 87 banded birds) at more than one site during the winter of 2006/2007, but none of these birds left their primary winter site permanently. We observed 2 migrants and 1 resident (total n = 260) at two sites during winter censuses conducted in November, January and March from 1999–2009; two birds returned to their original site of capture and one migrant changed wintering locations. Given the high winter site fidelity of both residents and migrants, we therefore believe that the absence of a detectable fecundity survival trade-off is most likely a consequence of differences in the quality of resident and migratory American dippers.

Conditional asymmetries among individuals may explain partial migration and differences in the fitness benefits of individuals that migrate or remain resident [50]. In our study population, American dippers that remain at lower elevations throughout the year initiate reproduction earlier [27], have higher annual productivity [28], and raise offspring that have higher juvenile survival than those produced by altitudinal migrants [29]. This study suggests residents also have slightly higher monthly apparent survival than migrants during all stages of the year, with annual apparent survival of residents estimated to be 5% higher than that of migrants. Altitudinal migration in American dippers is therefore likely to be an outcome of competition for the limited number of territories with suitable nesting habitat at lower elevations, with dominant individuals gaining access to higher quality territories at low elevations with less competitive individuals being forced to migrate to higher elevations in order to breed. Other studies suggest competition for critical resources on territories can influence whether individual birds migrate or remain resident. In European robins (*Erithacus rubecula*), dominant individuals monopolise high quality habitat with sufficient food to allow them to overwinter on their breeding grounds [51], and these sedentary individuals are more likely to attract a mate the following spring than migrants [26]. Field studies that demonstrate that migration is more prevalent at higher densities and/or when food is scarce also suggest a role for competition in explaining partial migration [22,52,53].

Seasonal patterns of mortality in migratory and sedentary individuals within partially migratory populations may differ simply because migrants have relatively high rates of mortality during migration and occupy different habitats from residents during one part of the annual cycle. We, however, found no evidence for differences in the timing of mortality for migratory and resident dippers; the model including a winter period*migratory strategy interaction term received half the support of the additive model (w_i_ = 0.08 compared to w_i_ = 0.16). Our data therefore suggest that mortality during migration is relatively low in this altitudinal migrant. In contrast, rates of mortality can be substantially higher on migration than during sedentary periods for some passerines and raptors that migrate long distances [12,13]. We estimated that winter monthly apparent survival was 2.4% lower than the apparent survival during the spring and summer when some dippers migrate and migrants and residents reproduce and molt. Winter survival is lower than survival at other stages of the annual cycle for a number of migratory birds (e.g. red-knot *Calidris canutus canutus* [54], houbara bustard *Chlamydotis undulata* [55], common pochard *Aythya ferina* [56]).

Winter survival of American dippers was influenced more by abiotic factors than the migratory behaviour of individuals. For both migrants and residents, monthly apparent survival during early and late-winter decreased as the maximum average daily flow rate declined. We also found some evidence that monthly apparent survival also decreased as the minimum daily temperature became more extreme. The latter result is consistent with a study examining variation in the annual survival of Eurasian dippers in southern Norway [33]. The former result is more counterintuitive as the annual survival of Eurasian dippers in France and the abundance of brown dippers (*Cinclus pallasii*) in Taiwan is lower in years when rivers flood [34,35,57]. In these studies flooding was argued to reduce the number, biomass and availability of invertebrate prey. Our result cannot be explained by an absence of winter flood events; the maximum daily flow rates in November 1999 (380 m^3^/s) and January 2002 (349 m^3^/s) approach the maximum flow rates on record [38]. One plausible explanation for the positive relationship between maximum daily flow rate and winter survival is that low maximum flow rates are associated with periods where mean flow rates are low reducing the amount of foraging habitat, and high maximum flow rates open up side channels and off-channel ponds that are preferred winter habitat for juvenile coho salmon *(Oncorhynchus kisutch)* and steelhead trout [58].

In summary, this study shows that migratory and resident American dippers have similar apparent survival rates during the migration-breeding period when they occupy different habitat and during the winter periods when they coexist at low elevations. Migrants therefore do not obtain a survival benefit from altitudinal migration and since they have lower annual productivity will have lower lifetime reproductive success. Altitudinal migration in American dippers is likely to be an outcome of competition rather than a consequence of benefits obtained from moving to areas with more food, and less predators or disease. Further studies that document the fitness consequences of alternative migratory strategies in partial migrants in a diversity of taxa are needed to shed light on the forces promoting migration.

## Supporting Information

S1 Dataset Mark-resighting dataset for migrant and resident American dippers in the Chilliwack River Valley from November 1999 to November 2009. (CSV)

S1 Table Model weighted parameter estimates of monthly survival for migrant and resident American dippers in the Chilliwack River Valley. Parameter estimates are provided for the period from November 1999 to November 2009 with unconditional standard errors and 95% Confidence Intervals.
(DOC)
