# Supplementary material for: Do American Dippers Obtain a Survival Benefit from Altitudinal Migration?
Source: PLoS One. 2015 Apr 23;10(4):e0125734. doi: 10.1371/journal.pone.0125734 (PMC4408061; doi:10.1371/journal.pone.0125734)
Supplement: S1 Table — Parameter estimates are provided for the period from November 1999 to November 2009 with unconditional standard errors and 95% Confidence Intervals. (DOC) [file pone.0125734.s002.doc]

Table S1. Model weighted parameter estimates of monthly survival for migrant and resident American dippers in the Chilliwack River Valley from Nov 1999 to November 2009 with unconditional standard errors and 95% Confidence Intervals.

|  | Migrants | | | | Residents | | | |
| --- | --- | --- | --- | --- | --- | --- | --- | --- |
| Time period | Phi | se | 95% CI | | Phi | se | 95% CI | |
| Nov 1999 – Jan 2000 | 0.931 | 0.021 | 0.878 | 0.962 | 0.936 | 0.017 | 0.895 | 0.962 |
| Jan – Mar 2000 | 0.934 | 0.021 | 0.876 | 0.966 | 0.936 | 0.015 | 0.899 | 0.960 |
| March – Nov 2000 | 0.947 | 0.010 | 0.924 | 0.963 | 0.952 | 0.009 | 0.932 | 0.967 |
| Nov 2000 – Jan 2001 | 0.929 | 0.019 | 0.881 | 0.958 | 0.934 | 0.015 | 0.898 | 0.958 |
| Jan – Mar 2001 | 0.930 | 0.027 | 0.855 | 0.968 | 0.933 | 0.021 | 0.879 | 0.964 |
| March – Nov 2001 | 0.946 | 0.009 | 0.924 | 0.962 | 0.952 | 0.008 | 0.932 | 0.966 |
| Nov 2001 – Jan 2002 | 0.931 | 0.021 | 0.875 | 0.963 | 0.936 | 0.018 | 0.892 | 0.963 |
| Jan – Mar 2002 | 0.935 | 0.022 | 0.873 | 0.967 | 0.937 | 0.016 | 0.896 | 0.963 |
| March – Nov 2002 | 0.947 | 0.009 | 0.925 | 0.962 | 0.952 | 0.008 | 0.933 | 0.965 |
| Nov 2002 – Jan 2003 | 0.930 | 0.019 | 0.881 | 0.959 | 0.935 | 0.015 | 0.898 | 0.959 |
| Jan 2003 – Mar 2003 | 0.933 | 0.021 | 0.877 | 0.965 | 0.936 | 0.014 | 0.901 | 0.959 |
| March – Nov 2003 | 0.947 | 0.009 | 0.925 | 0.962 | 0.952 | 0.008 | 0.933 | 0.966 |
| Nov 2003 – Feb 2004 | 0.927 | 0.023 | 0.866 | 0.961 | 0.932 | 0.020 | 0.882 | 0.962 |
| Feb – Mar 2004 | 0.932 | 0.025 | 0.862 | 0.968 | 0.935 | 0.019 | 0.886 | 0.964 |
| March – Nov 2004 | 0.947 | 0.009 | 0.925 | 0.962 | 0.952 | 0.008 | 0.933 | 0.966 |
| Nov 2004 – Jan 2005 | 0.930 | 0.023 | 0.871 | 0.964 | 0.936 | 0.019 | 0.888 | 0.964 |
| Jan – Mar 2005 | 0.930 | 0.028 | 0.851 | 0.969 | 0.933 | 0.022 | 0.875 | 0.965 |
| March – Nov 2005 | 0.946 | 0.010 | 0.924 | 0.962 | 0.952 | 0.008 | 0.932 | 0.966 |
| Nov 2005 – Jan 2006 | 0.928 | 0.021 | 0.874 | 0.960 | 0.934 | 0.017 | 0.891 | 0.961 |
| Jan – Mar 2006 | 0.939 | 0.022 | 0.876 | 0.966 | 0.936 | 0.015 | 0.899 | 0.961 |
| March – Nov 2006 | 0.947 | 0.010 | 0.924 | 0.963 | 0.952 | 0.008 | 0.933 | 0.966 |
| Nov 2006 – Jan 2007 | 0.928 | 0.021 | 0.876 | 0.959 | 0.934 | 0.017 | 0.893 | 0.960 |
| Jan – Mar 2007 | 0.930 | 0.028 | 0.852 | 0.969 | 0.933 | 0.022 | 0.875 | 0.966 |
| March – Nov 2007 | 0.946 | 0.010 | 0.924 | 0.962 | 0.952 | 0.009 | 0.932 | 0.966 |
| Nov 2007 – Jan 2008 | 0.930 | 0.022 | 0.873 | 0.963 | 0.936 | 0.018 | 0.890 | 0.963 |
| Jan – Mar 2008 | 0.930 | 0.027 | 0.857 | 0.968 | 0.933 | 0.021 | 0.880 | 0.961 |
| March – Nov 2008 | 0.947 | 0.010 | 0.925 | 0.963 | 0.952 | 0.008 | 0.933 | 0.966 |
| Nov 2008 – Jan 2009 | 0.930 | 0.021 | 0.875 | 0.963 | 0.936 | 0.018 | 0.892 | 0.992 |
| Jan – Mar 2009 | 0.929 | 0.028 | 0.851 | 0.968 | 0.935 | 0.017 | 0.894 | 0.987 |
| March – Nov 2009 | 0.946 | 0.010 | 0.923 | 0.963 | 0.952 | 0.009 | 0.931 | 1.000 |
